# Peptides actively transported across the tympanic membrane: Functional and structural properties

**DOI:** 10.1371/journal.pone.0172158

**Published:** 2017-02-24

**Authors:** Arwa Kurabi, Kerry A. Beasley, Lisa Chang, James McCann, Kwang Pak, Allen F. Ryan

**Affiliations:** 1 University of California San Diego, School of Medicine, Department of Surgery, Division of Otolaryngology, La Jolla, California, United States of America; 2 San Diego State University, School of Speech, Language, and Hearing Sciences, San Diego, California, United States of America; 3 San Diego Veterans Administration Medical Center, San Diego, California, United States of America; University of South Florida, UNITED STATES

## Abstract

Otitis media (OM) is the most common infectious disease of children under six, causing more antibiotic prescriptions and surgical procedures than any other pediatric condition. By screening a bacteriophage (phage) library genetically engineered to express random peptides on their surfaces, we discovered unique peptides that actively transport phage particles across the intact tympanic membrane (TM) and into the middle ear (ME). Herein our goals were to characterize the physiochemical peptide features that may underlie trans-TM phage transport; assess morphological and functional effects of phage peptides on the ME and inner ear (IE); and determine whether peptide-bearing phage transmigrate from the ME into the IE. Incubation of five peptide-bearing phage on the TM for over 4hrs resulted in demonstrably superior transport of one peptide, in level and in exponential increase over time. This suggests a preferred peptide motif for TM active transport. Functional and structural comparisons revealed unique features of this peptide: These include a central lysine residue, isoelectric point of 0.0 at physiological pH and a hydrophobic C-terminus. When the optimal peptide was applied to the TM independent of phage, similar transport was observed, indicating that integration into phage is not required. When 10^9^ particles of the four different trans-TM phage were applied directly into the ME, no morphological effects were detected in the ME or IE when compared to saline or wild-type (WT) phage controls. Comparable, reversible hearing loss was observed for saline controls, WT phage and trans-TM peptide phage, suggesting a mild conductive hearing loss due to ME fluid. Perilymph titers after ME incubation established that few copies of trans-TM peptide phage crossed into the IE. The results suggest that, within the parameters tested, trans-TM peptides are safe and could be used as potential agents for noninvasive delivery of drugs, particles and gene therapy vectors to the ME.

## Introduction

Otitis media (OM) is a multifactorial disease with widespread consequences. It is the most common infectious disease in children aged 6 months to 6 years, and is the most common illness-related reason for which a child will visit a doctor. This leads to a significant health care burden; estimated to result in more than 5 billion dollars each year. [1] Out of the many consequences of OM, permanent damage to the cochlea is one of the most severe. [2] However, transient conductive hearing loss due to persistent OM in children has been associated with language delays and cognitive development impairment. [1,3,4] Other serious complications that can arise from chronic OM include the development of mastoiditis and erosion of the mastoid cavity and middle ear (ME) walls. [5] OM is significantly more serious in the developing world, and undertreated OM is estimated to be responsible for one half of the world’s burden of serious hearing loss. [6,7]

Currently, the most common treatments for OM include systemic antibiotics and/or a surgical procedure involving myringotomy with insertion of a pressure-equalization tube (PET) through the tympanic membrane (TM). Systemic antibiotics frequently cause gastrointestinal and allergic side effects [8,9] and have been implicated as a promoter of antibiotic-resistant bacterial growth. [6,10] Additionally, the efficacy of using systemic antibiotics to treat OM is controversial. Pichichero and Reed [11] noted that no detectable amoxicillin was found in the ME fluid of 15–35% of children who received the drug systemically via oral administration. PETs have been shown to be an effective treatment option for chronic or recurrent OM, but the potential for later TM abnormalities, along with current uncertainty about the long-term efficacy of the treatment [12,13] make this therapy choice somewhat controversial. Furthermore, surgical procedures have risks associated with general anesthesia, which can be more serious in children. [14] Given that OM is extremely prevalent and costly, and that current treatment options have disadvantages, alternative avenues of treatment which overcome the limits of the current conventional therapies are needed

Local pharmacotherapy is a technique successfully used in the treatment of many diseases, including otitis externa. (e.g. [15]) Such local treatment for OM is difficult due to the TM barrier function. In both the human and rat, the outer TM epithelium consists of up to four cell layers of epidermal cells and is continuous with the lining of the external auditory canal (EAC), and an inner mucosal layer that has similar isolating properties, as it is continuous with the mucosal lining of the ME. The cells of these two layers are connected by tight junctions. In between these epithelial layers lies connective tissue consisting primarily of collagen fibers, which give the membrane the majority of its structural properties. The connective tissue layer of the rat and human TM are similar. [16] The TM consists of the pars tensa which transmits auditory vibrations to the manubrium of the malleus, and the smaller pars flaccida which is much less rigid and may play a lesser role in sound transmission. [16,17,18] Currently, crossing this anatomical barrier to provide localized pharmaceutical treatment to the ME can only be achieved clinically through physical penetration of the TM, via either injection by needle or PET insertion. Development of methods for local and noninvasive delivery of antibiotics to the ME would potentially decrease side effects, limit exposure of off-target bacteria, and increase the efficacy of treatment. Furthermore, if a simple-to-apply, local drug therapy was developed, outcomes of treatment in developing countries could potentially be improved. While penetrants have been used experimentally to enhance TM permeability and local antibiotic delivery across the TM, [19,20] enhanced diffusion methods are generally limited by the size of the cargo that can be delivered, which has led to the search for more specific, targeted forms of delivery. [21] A method for trans-TM delivery of a wide range of molecules, gene therapy vectors, particles and even bacteriolytic organisms to the ME would enhance the development of a range of novel therapies.

Phage display is a discovery method stemming from a novel approach which involves screening large numbers of biomolecule species on the surface of bacteriophage. [22] This method can be used to discover active transport mechanisms of barrier cells to identify specific peptide chains that can utilize any such mechanism that exists. [23] Recently, we screened a library of 10^12^ genetically engineered, random peptide-bearing M13 filamentous bacteriophage for the ability to cross the TM. Since nontypeable *Haemophilus influenzae* (NTHi) is now the most common cause of ME infection, and our goal is to provide a basis for clinically applicable improvements in pharmacotherapy, ME infection with NTHi was used during the phage selection process. Several small peptides (1000–2000 Da) were discovered that utilize an active transport mechanism to move phage particles across the intact TM. [24] The discovery of these peptides opens up the possibility that they could become a platform for trans-TM drug delivery, by linking to drugs or drug packages for local ME pharmacotherapy and treatment of OM. Additional possibilities include transport of gene therapy vectors and lytic phage specific for pathogens that mediate OM. However, a number of important issues remain to be resolved including effectiveness and safety. The features of the peptides that mediate trans-TM transport are unknown. Also, can the peptides cross the membrane without attachment to phage? Are trans-TM transport peptides safe? Do they target the ME without subsequent transport into the inner ear (IE)?

The purposes of this study were to compare the potency and physiochemical properties of five trans-TM phage particles at longer intervals than those tested previously; to determine whether an optimal trans-TM peptide phage can be identified based on transport characteristics; to determine whether trans-TM peptide can cross the membrane independent of phage; to explore the structural features of the peptides that are associated with optimal trans-TM transport; to evaluate the effects of peptide phage on the structure and function of the ME and IE; and to determine the extent to which trans-TM peptide phage may be transported into the IE.

## Materials and methods

### Animals

Young adult Sprague Dawley (SD) male rats, aged 60–90 days were used for all experiments. All research was performed according to the recommendations set forth by the National Institutes of Health (NIH) in the Guide for the Care and Use of Laboratory Animals. All experiments were conducted under a protocol approved by the Institutional Animal Care and Use Committee (IACUC) titled *Middle Ear Response in Otitis Media* (IACUC number A13-022), located at the VA San Diego Medical Center. Since phage transport takes place using an active transport mechanism, [24] experiments were conducted *in vivo*. In order to minimize the number of animals, both ears were utilized when possible. Experimental models for OM are commonly carried out in rats due to the structural and morphological similarities between rat and human TMs. [25,26] Anesthetic cocktail consisted of ketamine (40 mg/kg), xylazine (10 mg/kg), and acepromazine (0.75 mg/kg). The cocktail was administered through intraperitoneal (IP) injection at a dosage of 0.4 mL per 100 grams. Depth of anesthesia was measured by toe pinch at least every 15 minutes, and supplementary anesthetic doses were given as needed. The skin of the neck was prepped and disinfected with betadine solution. The surgical procedure was performed via midline neck incision and dissection of the soft cervical tissues to expose the ventral bullae bone. Using a sterile 25-gauge needle, a hole was carefully drilled in the ME bulla center with precautions taken to not damage any delicate ME structures or the TM.

### Phage transport screening and trans-TM transport rate evaluation

As noted above, utilizing phage display, we identified a number of peptides that are actively transported through the TM attached to bacteriophage. [24] To further assess comparative transport rates over time, individual bacteriophage bearing trans-TM peptides were exposed to the TM *in vivo*, 48 hrs after inoculation of NTHi into the ME through the ventral bulla bone, exposed via midline neck incision, to induce OM. Care was taken to avoid the area of the TM, and TMs were visually inspected after ME inoculation to determine that no perforation was present. Five different peptide-bearing phage particles were amplified in *E coli*, and 10^9^ phage particles in 50 μL of PBS were applied to one TM of six anesthetized rats per time point/peptide. After 1, 2 or 4 hrs, the external ears of the rats were rinsed repeatedly with PBS to remove any phage particles. The MEs of the rats were then isolated, and further rinsed as a precaution against any contamination. ME effusion was then harvested. The number of phage particles recovered in the fluid was titered accordingly. Lysogeny broth (LB) was inoculated with *E*. *coli* ER2738 host strain and the culture was incubated, with shaking, for 4–6 hrs. 3 mL of liquid top agar was prepared in culture tubes for each dilution and maintained at 42°C prior to use. 10 μL of each sample was serially diluted into a culture tube, vortexed briefly, and poured immediately onto plates containing LB, isopropyl beta-D-thiogalactopyranoside (IPTG), and 5-bromo-4-chloro-3-indolyl-β-D-galactopyranoside (X-gal). Clear blue plaques of dead bacteria on the LB/IPTG/X-gal plates were counted and multiplied by the dilution factor to convert titer results into plaque-forming units (pfu) per 10 μL. Phage titers recovered from the ME were compared to those observed for wild-type (WT) phage (not bearing a peptide) as negative controls.

### Trans-TM transport of free peptide

To assess the ability of peptides to cross the TM independent of phage, TM-3 peptide was synthesized and linked to a 150 bp DNA tag for qPCR analysis (BioSynthesis, TX, USA). Phage was then applied to the TMs of infected rats in the same manner described above for peptide phage. 50 nmoles of the labeled peptide in 50 μL of PBS was incubated on the TM for 1 hr. The ear canal was then extensively rinsed with PBS. The ME was opened and the contents harvested and subjected to qPCR using primers specific for the DNA tag. A standard curve was generated by amplifying dilutions of the labeled peptide. This curve was used to determine the amount of peptide present in each ME.

### Structural analysis of trans-TM peptides

To explore the potential basis of trans-TM transport as well as differences in transport efficiency, we analyzed the structure of all five peptides. We sought to identify unique features associated with a more efficient transport rate, and with exponential increases in transport over time that would be expected with an active transport mechanism that does not saturate. To accomplish this, we evaluated the influence of amino acid composition using Pepcalc software, [27] amino acid position, peptide primary structural features using Chemdraw (Perkin Elmer) and secondary and tertiary architecture, conjugated to M13 P3, using PepFold 3.0. [28]

### Phage peptide effects on ME and IE morphology

The ME bullae of uninfected rats were injected with 10 μL containing 10^9^ phage particles through a well-established surgical procedure and as described previously. [29–31] Following fenestration of the bullae, a 28-gauge insulin syringe was used to administer 10 μL of phage in experimental conditions, or 10 μL of saline in control animals. Sterile cotton swabs were used to remove any excess fluid from the exterior of the bulla following the injection. The injection sites were again covered with cervical soft tissues and efforts were made to restore the musculature of the area close to pre-operative positioning. Visual inspection of the TMs under microscope verified they were still intact after the completion of the procedure.

After a 24 hr ME phage incubation in uninfected rats, the animals were anesthetized and sacrificed through a cardiac perfusion. Following dissection, tissues were fixed in PFA for 24 hrs and then decalcified in 4% PFA and 8% ethylenediaminetetraacetic acid (EDTA) for 21 days. Ears were then paraffin embedded, cut into 10-μm sections, and mounted onto slides. The slides were then stained with hematoxylin and eosin so that the morphology of the ME and IE could be evaluated.

### Effects of trans-TM peptide phage on auditory function

To investigate possible effects of the peptides on ME and IE function, hearing sensitivity was tested prior to saline, WT phage or each of the four trans-TM phage peptides instillation into the ME, and then at 24, 48, and 72 hrs post-surgery. Pure tone ABR thresholds were obtained, using previously established methods [32–34] which generate thresholds that closely correspond to hearing sensitivity measured via behavioral testing. [35] Previously, it has been shown that anesthesia with ketamine yields ABR thresholds that are more stable over time than other anesthetics. [36] Accordingly, animals were deeply anesthetized using the same ketamine-based rodent cocktail described above. A Tucker-Davis-Technologies evoked potential Systems III running the BioSigRP program was utilized to measure ABR thresholds in response to tone-pips (5 ms, 0.5 ms ramp) delivered at a rate of 10 stimuli per second. Responses to stimuli for 8, 16, and 32 kHz were obtained at intensity levels descending from 90 to 5 dB SPL. Response recordings using a 10 msec time window were filtered and amplified, averages were made over 512 runs and the averaged traces were recorded. These frequencies were chosen because they make up the range most susceptible to damage from various causes. (e.g. [37,38]) Threshold was defined as the stimulus level between the lowest intensity tracing with a visible response and the highest intensity tracing with no detectable response. This was completed using an open speaker system in a sound-attenuating chamber. Subcutaneous needle electrodes were placed on the vertex (active electrode), below the pinna of interest (reference electrode), and on a hind limb of the animal (ground electrode). The response was collected simultaneously with stimulus onset, and then amplified and filtered by the Tucker-Davis Technologies system. This allowed all stimuli, recording, and averaging functions to be computer-controlled. Thresholds were obtained for the four peptide-targeted phage, as well as the WT phage and saline controls, prior to ME application and at each post-application day.

### Entry of trans-TM peptide phage into the IE

To examine the possibility of TM-transmigrating phage that enter the ME might further transmigrate into the IE, rat MEs were exposed to peptides *in vivo* for 4 hrs. The IEs were harvested and extensively rinsed. An opening was made in the scala tympani wall on the cranial side of the IE. Perilymph was then sampled, first with two glass micropipette capillaries per ear, and then by micro-syringes filled with 5 μl PBS. To determine whether phage particles were present in perilymph, titering was performed as described above.

### Statistical analysis

A two-way repeated measures analysis of variance (ANOVA-R) was completed using GraphPad® software. In order to investigate which means were statistically significant from one another, Tukey multiple comparisons post-hoc testing with Bonferroni correction was completed. Statistically significant was defined as differences between groups that reached a P value of <0.05. Confidence intervals were illustrated using standard error bars.

## Results

### One peptide mediated optimal trans-TM transport

**Fig 1:**
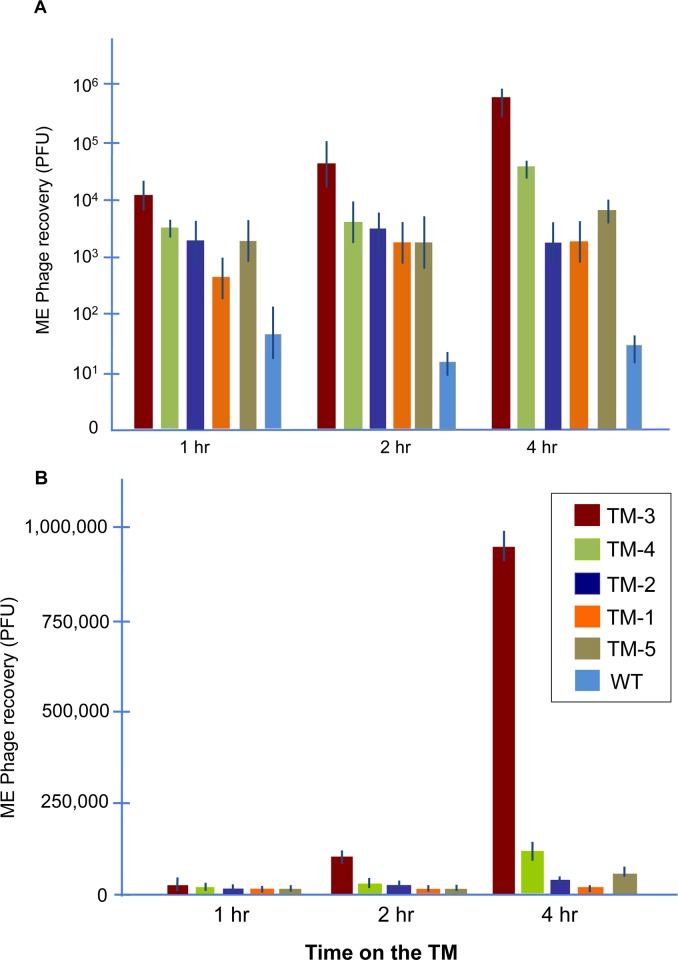
Kinetics of peptide-mediated trans-TM transport. ME recovery kinetics of five TM-transiting peptide phage, compared to WT phage particles, following incubation of 10^10^ phage on the exterior surface of the TM for 1, 2 or 4 hrs. **A** = log scale, **B** = linear scale. All TM-transiting peptide phage were recovered at levels much higher than were WT phage not bearing a peptide. Phage bearing TM-3 peptide were recovered at much higher titers (more than 10^5^ higher at 4 hrs) than WT phage, and significantly more than any other TM-transiting phage. TM-3 phage also exhibited exponential increases in ME recovery over time consistent with an active transport mechanism. Error bars = SEM (n = 6 animals).

The titers of five trans-TM peptide phage that were observed in the MEs of rats at 1, 2 or 4 hrs after application to the external surface of the TM are illustrated in Fig 1. Variation in phage transport across the TM was noted, with phage bearing peptide TM-3 showing markedly higher levels of transport. Phage entry into the ME increased exponentially as the duration of contact with the external surface of the TM was extended, consistent with an active transport process, for TM-3 and TM-4 phage. The remaining phage showed lower rates of increase over time, consistent with less efficient and/or saturated transport.

### Transport characteristics are associated with peptide structure

**Fig 2:**
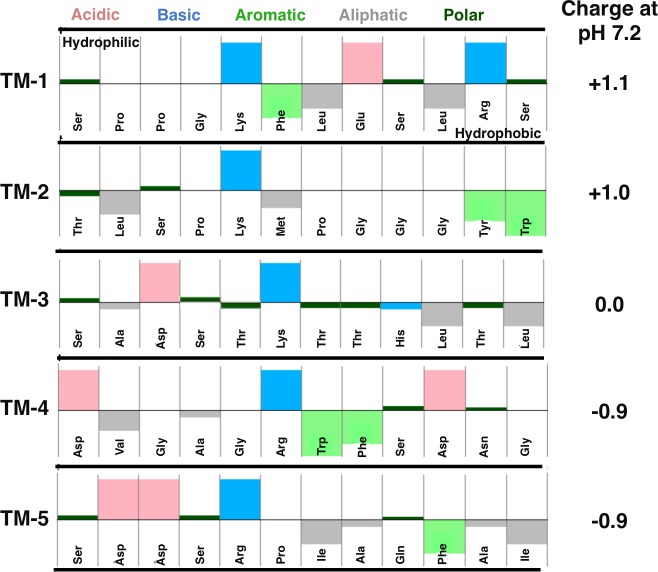
Composition analysis of trans-TM peptides. The peptides of five trans-TM phage exhibiting a range of TM transit efficiencies were evaluated. PEPCALC analysis of amino acid characteristics and hydrophility/hydrophobicity (**A**) and isoelectric properties (**B**).

To explore the potential basis of superior transport by peptide phage TM-3, we analyzed the structure of the five peptides, to identify unique features of TM-3 that might explain its more rapid trans-TM transport rate. We also sought to identify unique features of TM-3 and TM-4 that might explain their exponential rate of transport over time, when compared to the remaining selected peptides. Pepcalc analysis (Fig 2) indicated that the two peptides that exhibited exponential increases in transport over time (TM-3 and TM-4) were both characterized by a basic amino acid at position 6, and strongly hydrophobic residues at the C-terminus. In addition, our optimal peptide TM-3, based on transport rate, also was predicted to be neutral at physiologic pH (7.2). None of the other 4 peptides shared all of these characteristics.

**Fig 3:**
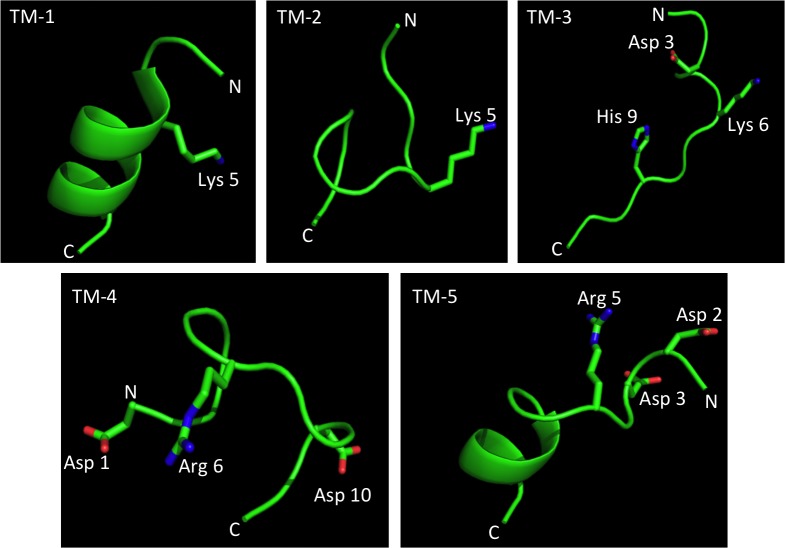
3-D structural analysis of the five trans-TM peptides. Structures were predicted using Pep-Fold 3.0. N = amino-terminus; C = carboxy terminus. Selected amino acids indicated by position relative to N.

When the peptides were analyzed for secondary and tertiary structure in their position relative to the N-terminus of the M13 P3 protein, significant differences in predicted 3-D structures were apparent (Fig 3). TM-1 and TM-5 contained αhelices, while. TM-2, TM-3 and TM-4 adopted the highly flexible random coil structure. Hence, these peptides can optimize their confirmation in solution upon ligand binding. The basic amino acid extensions (either arginine or lysine) at position 6 in peptides TM-3 and TM-4 were solution exposed and physically close to asparagine residues, but this was also true of TM-5. The histidine imidazole ring of TM-3 was a unique feature. In addition, TM-3 was rich in hydroxyl containing amino-acids serine and threonine.

### Free trans-TM peptides are transported into the ME

**Fig 4:**
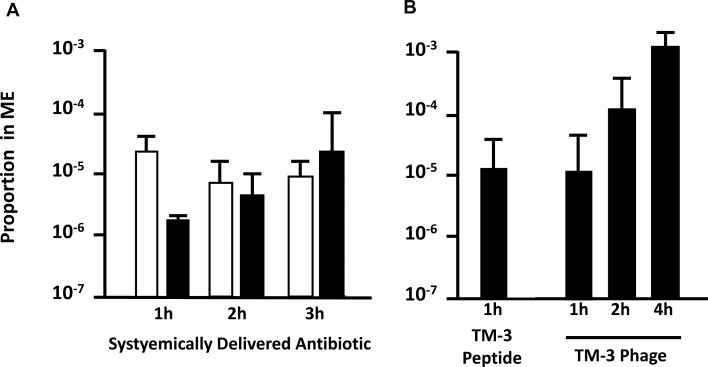
Comparison of peptide phage transport to systemic antibiotic delivery in the ME. **A.** Proportion of systemically delivered antibiotics that were observed in the ME of patients at 1, 2 or 3 hrs after systemic administration by Silverstein et al. [43] (penicillin, 500,000 units; black bars) or Nicolau et al. [44] (cefprozil, 15 mg/kg; white bars). **B.** Proportion of TM-3 peptide phage, applied to the TM, that was recovered from the ME 1, 2 or 4 hrs later, and TM-3 peptide, applied to the TM, recovered from the ME 1 hr later. Error bars = SEM (n = 4).

When DNA-tagged TM-3 was applied to the TM as a free peptide for 1 hr, qPCR detected free peptide in the ME. The proportion of peptide transported was similar to the proportion observed for TM-3 phage, indicating that transport is independent of peptide linkage to phage (Fig 4).

### TM-transiting peptide phage have no effect on external ear, TM, ME and IE morphology

**Fig 5:**
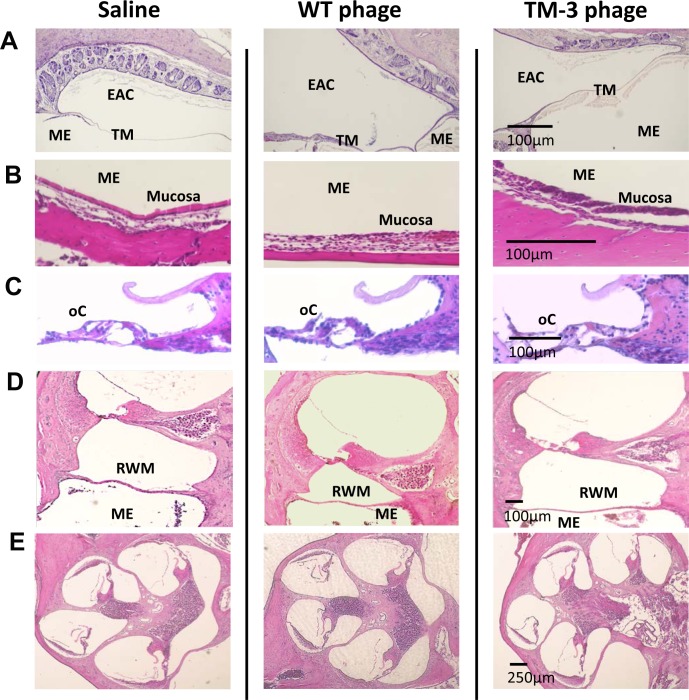
Histological evaluation of the ear after trans-TM peptide phage application, compared to WT phage negative control and saline control. **A.** Hematoxylin and eosin (H&E) stained sections illustrate the external auditory canal (EAC), TM and ME, 24 hrs after application of 10^10^ TM-3 phage or controls to the external surface of the TM. **B-E.** The ME and ME mucosa (**B**); the organ of Corti (C); the ME, RWM and cochlear basal turn (**D**) and the cochlea (**E**), 24 hrs after application of 10^10^ TM-3 phage directly into the ME.

No significant differences were observed in ME histology when comparing the peptide phage groups to each other, or when they were compared to the two control groups. The external ear epithelium, TM, ME lumen, and ME epithelium all appeared normal and remained intact, as illustrated in Fig 5. As also shown, the IE including the round window membrane (RWM), organ of Corti, spiral ganglion, stria vascularis and auditory nerve were normal in appearance.

### TM-transiting peptide phage have no effect on auditory thresholds

**Fig 6:**
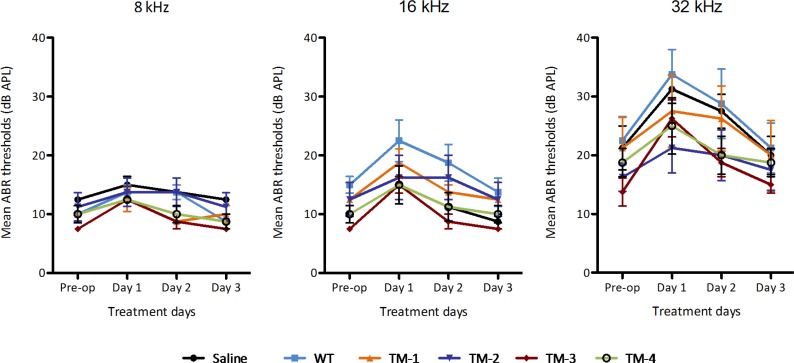
Impact of trans-TM peptide phage on hearing. ABR thresholds measured in dB SPL (Sound Pressure Level relative to .0002 dynes/cm^2^) were recorded pre-operatively and daily for four days following 10^10^ phage particle injection into the ME bulla. Responses were obtained for 8, 16, and 32 kHz pure-tone stimuli. Results indicate that thresholds were elevated up to 20 dB on postoperative days 1 and 2, recovered to within +/- 5 dB of baseline by day 3. Results for four TM-transiting phage, WT phage and saline were similar, indicating that the temporary change in threshold is due to fluid, and that no damage to hearing occurred as a result of TM-transiting phage application. Data are means + SEM (n = 8).

ABR testing at 8, 16, and 32 kHz prior to and after ME application revealed no significant differences in the effect of any type of treatment—including all four peptide phage treatments as well as both control treatments—and no significant interactions between treatment type and day post treatment results (Fig 6). A significant effect of day post-treatment was observed (<0.0001) at each frequency tested, as seen in Fig 2. Post-hoc testing utilizing Tukey multiple comparisons revealed significantly reduced hearing sensitivity when comparing day 1 results to pre-operative thresholds (<0.0001) at all three frequencies and for all treatment groups, including the WT phage and saline control groups. No significant difference between treatment groups was noted on any given post-treatment day.

As seen in Fig 6, significantly worse hearing sensitivity was seen on day 1 vs. day 2 (0.0032), day 1 vs. day 3 (<0.0001), and day 2 vs. day 3 (0.0104) for 8 kHz. No significant difference was seen between pre-operative results compared to day 2 or day 3 thresholds. 16 kHz revealed significantly worse hearing sensitivity when comparing pre-operative results to day 2 (0.0370), day 1 vs. day 2 (<0.0001), day 1 vs. day 3 (<0.0001), and day 2 vs. day 3 (0.0083). No significant difference was seen between pre-operative thresholds and thresholds on day 3 indicating that hearing thresholds returned to basal pre-experimental levels.

### TM-transiting phage showed minimal penetration into the IE

**Fig 7:**
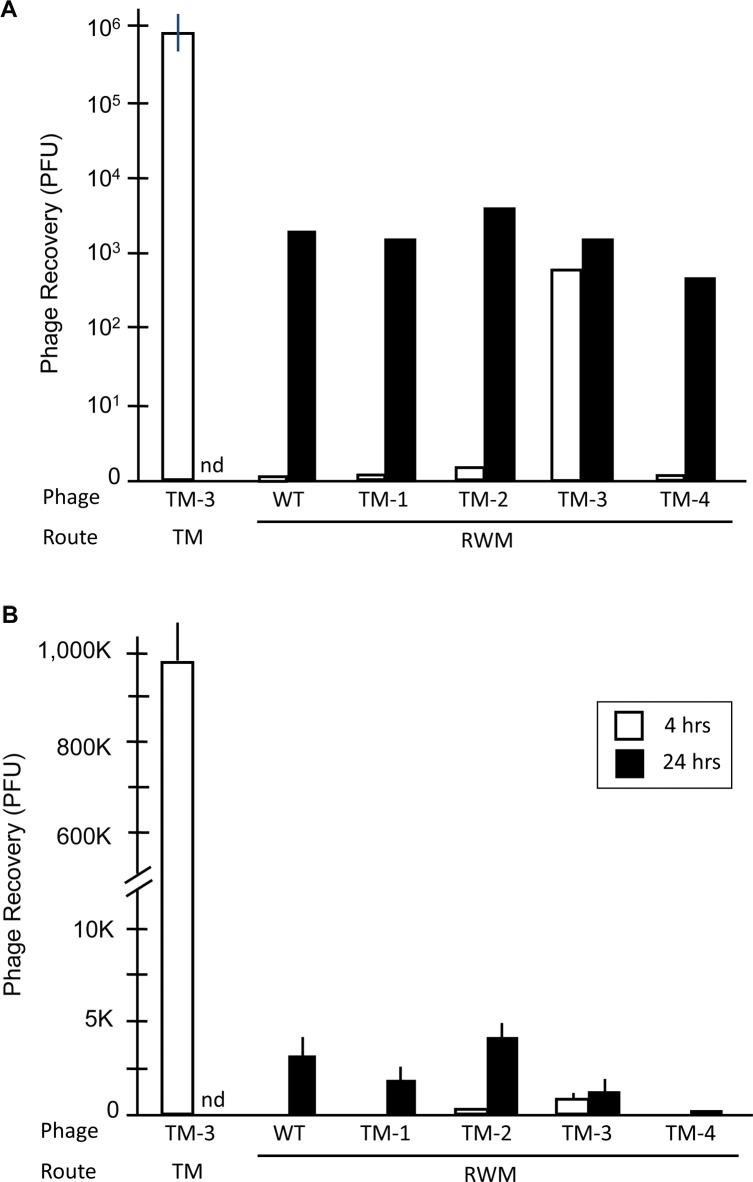
Comparison of trans-TM peptide entry into the IE versus the ME. Compared to ME recovery of TM-3 after 4-hr application of 10^9^ phage to the TM (white bar, taken from Fig 1), recovery of TM-transiting phage from perilymph, 4 hrs after instillation of 10^9^ phage onto the area of the RWM (black bars) is absent or very low. Phage recovery from the IE improved somewhat at 24 hrs, but was not greater for TM-transiting phage than for WT Phage not bearing a peptide. Thus, any movement of phage into the IE does not appear to be peptide-mediated. Error bars = SD (n = 4).

The titers of perilymph revealed that no or very few (an average of 0–67) peptide-bearing phage were observed in the IE at 4 hrs after placement of 10^9^ phage in the area of the RWM, for WT, TM-1, TM-2 and TM-4. These results are compared to transit of TM-3 through the TM at the same exposure period in Fig 7. For TM-3, an average of 890 particles was recovered. This compares to nearly 10^6^ particles recovered 4 hrs after application of 10^9^ TM-3 phage to the TM. The figure also shows that one day after placement into the ME, from 600 to 4000 phage particles were recovered from perilymph for all phage, including WT and TM-3, approximately 10^6^ less than the titer applied to the RWM. These results are consistent with a low level of primarily passive transit, or perhaps small amounts of contamination during the IE fluid sampling procedure.

## Discussion

The existence of an active transport mechanism through the TM is surprising. The mechanisms must operate through epithelial cells of opposite polarity and through the connective tissue of the middle layer of the membrane. Whether transport occurs via the pars tensa, the pars flaccida or both is not clear. The differences between the connective tissue layer of the two parts of the TM would presumably play a limited role, since the collagen bundles that make up this layer are separated by sufficient space to allow penetration by particles the size of bacteriophage. [39]

It is clear from Fig 1 that phage TM-3 exhibited by far the highest level of trans-TM transport, and also increased exponentially over time. This provides additional evidence that its mechanism of transport is active, reinforcing our prior observations of temperature and oxygen sensitivity. [24] TM-4 peptide phage, which exhibited the next highest level of transport, also tended toward exponential increase over time. However, the remaining three peptides showed more modest increases in TM transit over time, indicating less efficient transport and possibly saturation.

When the composition and structure of the five trans-TM peptides were compared, no clear conformational pattern emerged. The most consistent feature was a basic lysine or arginine in the central position. For TM-3 and TM-4, the most efficient peptides, this amino acid was at position six. However, given the many peptides in the parent library that would have shared this feature and yet were not enriched, this cannot have been a defining feature. Another feature of TM-3 not shared by any of the other peptides is the presence of 6 serine and threionine residues. With 20 amino acids, the expected average number of serine or threionine in a 12-mer peptide is 1.2 (an average diversity of 0.68 per position). However, it is not clear how the hydroxyl containing amino acids are playing a role. Similarly, when 3-D structure was compared to the efficiency of trans-TM transport of phage bearing them, no particular fold emerged. TM-3 and TM-4 showed an irregular coil structure, as did the less efficient TM-2. Meanwhile, TM-1 and TM-5 adopted an ordered helical secondary structure. The large differences in amino acid sequence composition and structure of the five trans-TM peptides could argue against a unitary ligand/receptor model for transport and a unifying single uptake mechanism. Each peptide may induce its own unique combination of molecular affects and interactions. One possibility consistent with our data is multiple binding sites that mediate a pinocytotic trans-cellular transport event. Further studies will be required to understand the mechanism(s) of peptide-mediated transport into the ME through the intact TM.

TM-3 as a free peptide showed trans-TM transport that was equivalent in magnitude to TM-3 phage conjugate. This result indicates that the transport mechanism is not dependent upon attachment to phage cargo, but is determined by the TM-3 peptide itself. The fact that peptide and peptide phage transit rates were similar also implies that trans-TM transport is not influenced by the size of the transported molecular or particle. While the mechanism of active trans-TM transport is not known, the possibilities include a specific transmembrane transporter or vesicular transport within the cells of the TM, or alteration of para-cellular tight junctions allowing movement between cells. [21] It seems likely that a transporter or movement between cells via tight junctions would be sensitive to cargo size. This further implicates trans-cellular movement within vesicles, perhaps the most likely mechanism for active trans-TM transport. In this case, those unique structural features of peptide TM-3 as noted above might confer more efficient binding to a receptor or other membrane protein that induces vesicle formation, although this is clearly a speculation on our part.

The hearing sensitivity of SD rats showed statistically significant threshold shifts at 24 hrs post that resolved by 72 hrs after ME application in saline controls, WT phage controls, and all 4 peptides phage (TM-1-4). These threshold shifts occurred across the frequency range tested and were comparable in magnitude for all groups. As there were no significant differences between treatments, the hearing sensitivity findings suggest a slight, presumably conductive, hearing loss due to the surgical procedure and/or the presence of the fluid injected into the ME as a carrier for the phage, or as a control. At 8 kHz, no significant difference was seen between pre-operative results compared to day 2 or day 3 thresholds, suggesting 8 kHz hearing sensitivity recovers to pre-operative status by day 2. For 16 and 32 kHz, no significant differences were seen between pre-operative thresholds and thresholds on day 3, thus sensitivity at these frequencies recovered to pre-operative status by day 3. The transient hearing loss primarily at the highest frequencies is consistent with temporary mass damping due to the fluid injected into the ME, which may have altered the impedance matching between the ear canal, ME and IE. Alternatively, tissue edema or inflammation due to surgery could have played a role in the temporary shift in hearing sensitivity. However, no visible signs of inflammation or tissue swelling were observed in sections of the ME or IE (Fig 5), making this alternative unlikely.

Our results indicate that peptide phage TM-1, TM-2, TM-3 and TM-4 are not harmful to the structure or function of both the ME and IE systems at the time points that were examined. These findings have important implications for the potential of transtympanic peptides as drug chaperones. Obviously, they can only be investigated as tools for local drug delivery to the ME in humans, once proven safe in animal studies. It is also important that minimal transit of the RWM was observed. This suggests that any off-target delivery of drugs intended for the ME would be minimal. These are critical steps in the potential therapeutic development of TM-transiting peptides.

Very few TM-transiting phage entered the IE. It has been well documented that molecules can passively diffuse across the RWM during OM, including both bacterial substances such as endotoxin and host pro-inflammatory mediators. [40] However, M13 bacteriophage are much larger than such molecules, being approximately 0.9 μm in length. [41] Thus, it is not surprising that the level of entry of WT phage is minimal. However, since the ME mucosa lining the round window and the TM are presumably very similar, one might expect TM-transiting phage to also cross from the ME into the IE. However, the levels of IE phage transit observed are low, and too low to be explained by the difference in the area of the RWM versus the TM, approximately 65 to 1. [42] Also, while the transit of TM-3 at 4 hrs was somewhat higher than for other phage, transit did not increase at 24 hrs, which is inconsistent with efficient active transport. The apparent lack of active IE phage transit suggests either that the orientation of ME mucosal cells is critical to transtympanic transit, or that the mesenchymal cells that form the inner layer of the RWM block transit from the ME into the IE.

With respect to peptide-mediated TM transit, if successfully developed the active transport mechanism described here could be used to deliver a wide variety of therapies to the ME. This could include small and large molecules, gene therapy vectors, antimicrobial peptides (AMPs), and particles up to the size of M13 bacteriophage (as noted above, ~0.9 μm). [41] In addition, the method could also be used to deliver bacteriophage. There is increased interest in the use of lytic phage in infection control. They have an advantage over antibiotics that they are self-replicating, so that very small initial numbers will expand to eliminate bacteria in the numbers that are present. In addition, bacteriophage by their nature can avoid bacterial resistance, since they themselves can evolve along with their hosts.

## Conclusions

In summary, we have identified and utilized a natural cellular mechanism that mediates the active transport of large particles across the TM barrier. Several peptides with varying ability to transit the TM were identified, with rather variable sequences and predicted structures. Entry of two of these peptides increased exponentially over time, suggestive of efficient active transport. There are also no indications that peptides or phage are harmful to the external, ME or IE, or that TM-transiting peptide phage enter the IE from the ME in substantial quantities. Also, no damaging effects on hearing sensitivity were observed. The discovered peptides present a possibility for developing a local solution for ME pharmacotherapy via attachment of drugs, drug packages, gene therapy vectors or lytic phage that target OM pathogenic bacteria. This would provide a welcomed alternative to current treatment options, reducing exposure of off-target bacteria to antibiotics, side effects from systemic drug or vector delivery, as well as surgical risks. Furthermore, a noninvasive strategy for locally treating ME disease could prove to be extremely beneficial in developing countries where access to advanced medical treatment is often very limited. If simple to apply, this strategy could provide a feasible way to reduce acquired hearing loss and other serious complications due to under-treated OM. The data presented here provide evidence that this method could safely enhance local delivery of macromolecules and other therapeutics.
